# RETRACTION: Myosin 1b Participated in the Modulation of Hypoxia/Reoxygenation‐Caused H9c2 Cell Apoptosis and Autophagy

**DOI:** 10.1155/ancp/9869070

**Published:** 2026-04-28

**Authors:** Analytical Cellular Pathology

RETRACTION: J. Xu, J. Huang, X. He, M. Hu, S. Su, and P. Liu, “Myosin 1b Participated in the Modulation of Hypoxia/Reoxygenation‐Caused H9c2 Cell Apoptosis and Autophagy,” *Analytical Cellular Pathology*, 2022, 5187304, https://doi.org/10.1155/2022/5187304.

The above article, published online on 22 November 2022 in Wiley Online Library (wileyonlinelibrary.com), has been retracted by agreement between the journal Editor‐in‐Chief, Dimitrios Karamichos; and John Wiley & Sons Ltd. The retraction has been agreed due to concerns raised with the data presented in figures, some of which were identified on PubPeer [1] and involve the methodology of differentiating apoptotic cells from necrotic cells. Further investigation by the publisher noted additional inconsistencies. A summary of the additional concerns are below:

• The control flow cytometry plot for Figure 1a appears highly similar to the control flow cytometry plot shown in Figure 4b
• The HR panels in Figure 1d appear highly similar to the siRNA Wnt5a+LPS+Blank Serum panels in Figure 10 of [2]
• The si‐NC panels in Figure 4a appear highly similar to the siRNA Wnt5a+LPS+Serum 2 panels in Figure 10 of [2]

After assessment of the concerns and the author’s response by the editorial board, the results and conclusions can no longer be considered reliable, and therefore the article is retracted.

The authors agree to the retraction.
